# Proposal to change the name *Rhodoligotrophos* Fukuda *et al.* 2012, 1947 to *Rhodoligotrophus*. Request for an Opinion

**DOI:** 10.1099/ijs.0.056226-0

**Published:** 2013-09

**Authors:** Aharon Oren, George M. Garrity, Bernhard Schink

**Affiliations:** 1 The Institute of Life Sciences, The Hebrew University of Jerusalem, 91904 Jerusalem, Israel; 2 Department of Microbiology & Molecular Genetics, Biomedical Physical Sciences, Michigan State University, East Lansing, MI 48824-4320, USA; 3 Fachbereich Biologie, Universität Konstanz, D-78457 Konstanz, Germany

## Abstract

In the opinion of the authors, the genus name *Rhodoligotrophos* was formed in violation of Principle 3 and Rule 10a of the International Code of Nomenclature of Prokaryotes which requires that genus names are to be treated as Latin substantives. We therefore propose renaming the genus *Rhodoligotrophos* as *Rhodoligotrophus*. A Request for an Opinion is submitted to the Judicial Commission of the International Committee on Systematics of Prokaryotes regarding this proposed name change.

The genus name *Rhodoligotrophos* Fukuda *et al.* 2012, 1947 was published in 2012 with the following etymology: Gr. n. *rhodon* the rose; Gr. adj. *oligos* little, few; Gr. masc. or fem. n. *trophos* feeder, rearer, that which nourishes; N.L. masc. n. *Rhodoligotrophos*, red utilizer of few substrates (Fukuda *et al.*, 2012). In our opinion this name was formed in violation of Principle 3 and Rule 10a of the International Code of Nomenclature of Prokaryotes – The Bacteriological Code (Lapage *et al.*, 1992), which respectively state: ‘The scientific names of all taxa are Latin or Latinized words treated as Latin regardless of their origin. They are usually taken from Latin or Greek’, and ‘The name of a genus … may be taken from any source and may even be composed in an arbitrary manner. It is treated as a Latin substantive’. A few Greek substantives, including some that end with -os, have indeed been adopted in classical Latin without changing the ending to fit into one of the declensions of Latin nouns. This, however, does not make the genus name *Rhodoligotrophos* a name treated as Latin as intended by the Code. We therefore propose correcting the genus name to *Rhodoligotrophus* (Rhod.o.li.go.tro′phus. Gr. n. *rhodon* the rose; Gr. adj. *oligos* little, few; Gr. masc. or fem. n. *trophos* feeder, rearer, that which nourishes; N.L. masc. n. *Rhodoligotrophus* red utilizer of few substrates) in accordance with Principle 3 and Rule 10a of the Code.
